# Metabolomic Profiling of *Pogostemon cablin* Reveals Disruption of Secondary Metabolite Biosynthesis Induced by *Corynespora cassiicola* Infection

**DOI:** 10.3390/ijms26083680

**Published:** 2025-04-13

**Authors:** Ru-Xing Liao, Yang-Yang Chen, Li-Min Li, Ruo-Ting Zhan, Yu-Fan Chen

**Affiliations:** 1 Research Center of Chinese Herbal Resource Science and Engineering, School of Pharmaceutical Sciences, Guangzhou University of Chinese Medicine, Guangzhou 510006, China; 2 Key Laboratory of Chinese Medicinal Resource from Lingnan (Guangzhou University of Chinese Medicine), Ministry of Education, Guangzhou 510006, China

**Keywords:** patchouli, metabolomics, corynespora leaf spot disease, terpenoid biosynthesis

## Abstract

*Pogostemon cablin* (patchouli) is an economically important aromatic plant widely used in the fragrance and pharmaceutical industries. This study investigates the effects of Corynespora leaf spot disease (CLSD) on the metabolic profiles and patchouli alcohol content of patchouli leaves. Utilizing gas chromatography-mass spectrometry (GC-MS), real-time PCR (qPCR), and comprehensive non-targeted metabolomic analyses (HS-SPME-GC-MS and LC-MS/MS), we compared diseased (LD-TJ) and healthy (CK) leaves. Results revealed a significant 51% reduction in patchouli alcohol content in CLSD-infected leaves, which was correlated with a 94% decrease in expression of the patchoulol synthase (PTS)-encoding gene (*p* < 0.01) and a 79% reduction in farnesyl pyrophosphate synthase (FPPS)-encoding gene expression (*p* < 0.05), both critical for terpenoid biosynthesis. Metabolomic analyses identified extensive disruptions in both volatile and non-volatile compounds, with the majority of differential abundance metabolites (DAMs) being downregulated. Key metabolic pathways, including beta-alanine metabolism and nicotinate/nicotinamide metabolism, were notably affected, indicating broader metabolic instability. Additionally, crucial transcription factors involved in terpenoid biosynthesis were significantly downregulated, indicating a potential mechanism by which *C. cassiicola* may compromise patchouli quality through modulation of host metabolic processes. These findings underscore the urgent need to develop disease-resistant *P. cablin* cultivars through genetic and metabolic engineering to enhance the sustainability and productivity of this valuable industrial crop.

## 1. Introduction

Plant health is fundamental to sustainable agriculture and global crop security, playing a crucial role in maintaining ecosystem stability and human well-being [1,2,3]. Recent estimates suggest that plant diseases alone cause annual global crop losses of 10–16% during harvest and 6–12% in postharvest, threatening crop security and agricultural sustainability [4]. Among various plant diseases, fungal pathogens pose significant challenges to crop production systems worldwide. Corynespora leaf spot disease (CLSD), caused by the fungal pathogen *Corynespora cassiicola*, has emerged as a significant threat to global agriculture, affecting over 530 plant species across diverse genera [5]. This highly adaptable ascomycete thrives in tropical and subtropical climates, as well as in greenhouse environments, posing a persistent challenge for farmers worldwide [6,7]. The disease manifests with distinctive symptoms, including dark-brown necrotic lesions on leaves, often surrounded by yellow halos. These can expand into irregular or target-like patterns, leading to reduced photosynthetic capacity, premature defoliation, and significant yield and quality losses [8]. In rubber tree plantations, for instance, it is considered one of the most damaging diseases, causing severe defoliation and reduced latex production [9]. With changing climate patterns and intensive agricultural practices, the management of plant diseases such as CLSD has become more challenging, necessitating comprehensive studies to understand their effects on crop quality and productivity [3].

Metabolomics has emerged as a powerful analytical approach in modern plant science, offering comprehensive insights into the complex biochemical responses of plants to various environmental stresses and pathological conditions [10,11]. Hence, this technology essentially has two strategies: targeted and untargeted approaches [12]. Targeted metabolomics quantifies a particular set of predefined polar and/or nonpolar compounds that are already known to be present in the preparation [13]. In contrast, global untargeted approaches are more suitable for plant research, as they can identify thousands of metabolites (including novel ones) without prior knowledge, thereby providing opportunities for biomarker discovery or identifying unexpected metabolic changes [14]. The application of untargeted metabolomics in plant pathology has been especially useful in assessing plant-pathogen interactions and the implications on crop quality [15]. For instance, metabolomics analysis of *Arabidopsis* revealed how metabolome reconfiguration modulates plant immunity against diverse pathogens, including *Pseudomonas syringae* and *Botrytis cinerea* [16]. Using untargeted UHPLC-MS/MS metabolomics analysis of soybean plants during *Phakopsora pachyrhizi* infection revealed significant metabolic reprogramming in phenylpropanoid and terpene pathways [17]. Through untargeted metabolomics analysis, it was revealed that *Fusarium solani*, a pathogenic fungus, compromises the medicinal quality of *Panax ginseng* by significantly reducing the accumulation of saponins [18]. In conclusion, untargeted metabolomics offers a powerful approach to understanding the complex interplay between plants and pathogens, providing valuable insights into the impact of infections on crop quality.

*Pogostemon cablin* (Blanco) Benth., commonly known as patchouli, is a cornerstone species in the global pharmaceutical and fragrance industries. In traditional medicine systems, particularly Traditional Chinese Medicine (TCM), patchouli has been extensively utilized for its therapeutic properties, including anti-inflammatory, antidepressant, and antimicrobial activities, primarily attributed to its essential oil rich in volatile sesquiterpenes [19,20,21]. Additionally, patchouli contains a variety of non-volatile compounds such as flavonoids, organic acids, alkaloids, and phenylpropanoids, which exhibit numerous biological activities, including anti-inflammatory and anticancer effects [22,23,24]. In *P. cablin*, essential oils constitute approximately 1.5% of the plant’s composition, with patchouli alcohol (PA) accounting for nearly 30% of this fraction [25]. PA serves as the standard chemical marker for quality control of Pogostemonis Herba in the Chinese Pharmacopoeia, which comprises the dried aerial parts of *P. cablin* [26]. The biosynthesis of PA is organelle-specific, with leaf concentrations being eight to ten times higher than those in stems and flowers [27]. The PA biosynthetic pathway involves three critical stages: the mevalonate (MVA) pathway, farnesyl pyrophosphate synthase (FPPS), and patchoulol synthase (PTS) [28]. The MVA pathway initiates PA biosynthesis from acetyl-CoA. FPPS condenses dimethylallyl diphosphate (DMAPP) with two isopentenyl diphosphate (IPP) molecules to form FPP, which is subsequently cyclized to PA by PTS. Regulatory networks involving PatJAZ6, PatSWC4, PcbZIP44, PatDREB, and PcENO3 coordinately modulate PTS expression [29,30,31,32]. As a vital economic crop, patchouli is predominantly cultivated in tropical and subtropical regions, especially in Indonesia, Malaysia, China, and India. However, its cultivation faces significant challenges due to susceptibility to various phytopathogens, including multiple foliar diseases, among which CLSD poses a severe threat and has been reported in Guangdong and Hainan provinces, the primary patchouli production regions in China [33,34].

The objective of this study was to evaluate the effects of CLSD on the metabolic profiles and PA content of *P. cablin* leaves. Specifically, the research aimed to compare the PA content between healthy and CLSD-infected leaves and to analyze the corresponding changes in the expression of genes involved in terpenoid biosynthesis pathways. Additionally, the study conducted comprehensive non-targeted metabolomic analyses of both volatile and non-volatile compounds to elucidate the broader metabolic disruptions induced by CLSD. By elucidating these molecular and metabolic alterations, the study seeks to provide insights into the mechanisms by which *C. cassiicola* compromises patchouli quality, thereby informing strategies for developing disease-resistant cultivars and enhancing the sustainability and productivity of this valuable industrial crop.

## 2. Results

### 2.1. CLSD Significantly Reduced Patchouli Alcohol Content in Leaves

**Figure 1:**
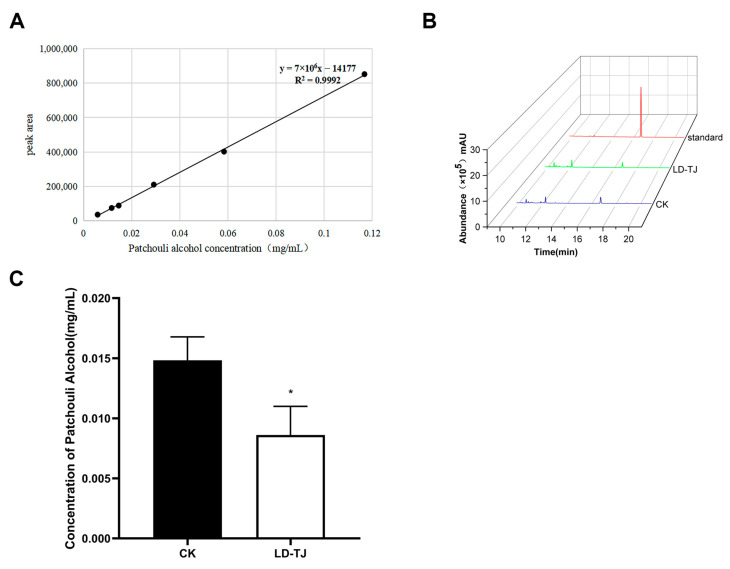
GC-MS analysis of patchouli alcohol in *Pogostemon cablin*. (**A**) The standard curve of patchouli alcohol standard. (**B**) GC-MS chromatograms of patchouli alcohol standard, healthy (CK) and diseased leaves (LD-TJ). (**C**) Concentration of patchouli alcohol in healthy (CK) and diseased leaves (LD-TJ). *, *p* < 0.05 (the *p*-values were obtained from the Student’s *t*-test). Each experiment was biologically repeated three times.

Given the importance of PA as a benchmark compound for evaluating the quality of Pogostemonis Herba, we performed gas chromatography-mass spectrometry (GC-MS) analyses on both *C. cassiicola*-inoculated (LD-TJ) and healthy (CK) leaves to identify variations in PA content. Employing the detection method established in preliminary studies, we constructed a standard curve for PA (Figure 1A). The measurement results indicated the presence of PA in LD-TJ and CK leaves, with a retention time of 14.91 min (Figure 1B). Quantitative statistical analysis revealed a significant reduction of 51% in the PA content in diseased leaves compared to healthy leaves (Figure 1C).

### 2.2. CLSD Significantly Reduced the Expression Level of Patchouli Alcohol Biosynthesis Pathway Genes

**Figure 2:**
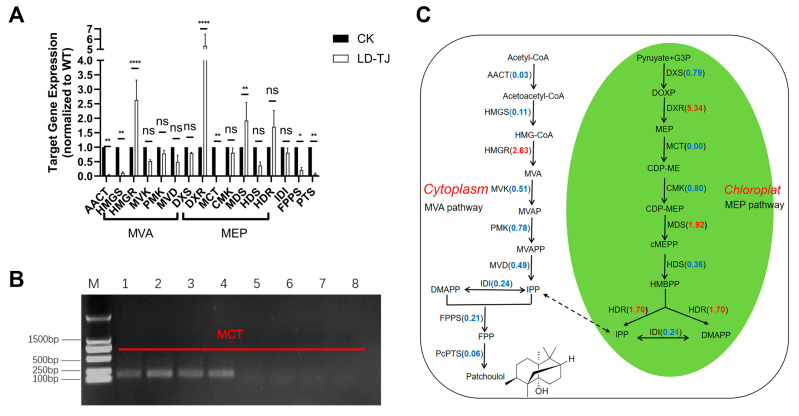
Expression profile levels of genes involved in the terpenoid biosynthesis pathways. (**A**) Relative gene expression assessed by RT-qPCR analysis. Asterisks indicate significance compared to CK controls, which were set to a value of 1. ****, *p* < 0.0001; **, *p* < 0.01; *, *p* < 0.05; ns, not significant, *p* > 0.05 (by two-way ANOVA with Bonferroni’s multiple-comparison test, n ≥ 3 independent experiments performed in triplicate). (**B**) Gene expression analysis of MCT by RT-PCR. M represents the DL2000 marker bands, lanes 1–4 represent the expression levels of the MCT protein-encoding gene in four biological replicates of the CK group, and lanes 5–8 represent the expression levels of the MCT protein-encoding gene in four biological replicates of the LD-TJ group. (**C**) Gene expression values are normalized to the control group (CK), shown as an average of three replicates, with blue numbers representing downregulation and red numbers representing upregulation. Abbreviations: AACT, acetyl-CoA C-acetyltransferase; HMGS, hydroxymethylglutaryl-CoA synthase; HMGR, hydroxymethylglutaryl-CoA reductase; MVK, mevalonate kinase; PMK, 5-phosphomevalonate kinase; MVD, mevalonate pyrophosphate decarboxylase; DXS, 1-deoxy-D-xylulose 5-phosphate synthase; DXR, 1-deoxy-D-xylulose 5-phosphate reductoisomerase; MCT, 2-C-methyl-D-erythritol 4-phosphate cytidylyltransferase; CMK, 4-(cytidine 5′-diphospho)-2-C-methyl-D-erythritol kinase; MDS, 2-C-methyl-D-erythritol 2,4-cyclodiphosphate synthase; HDS, 4-hydroxy-3-methylbut-2-enyl diphosphate synthase; HDR, 4-hydroxy-3-methylbut-2-enyl diphosphate reductase; IDI, isopentenyl diphosphate isomerase; IPP, isopentenyl diphosphate; DMAPP, dimethylallyl diphosphate; FPPS, farnesyl diphosphate synthase; PcPTS, *P. cablin* patchoulol synthase. G3P, glyceraldehyde 3-phosphate; DOXP, 1-deoxy-D-xylulose 5-phosphate; MEP, 2-C-methyl-D-erythritol 4-phosphate; CDP-ME, 4-(cytidine 5′-diphospho)-2-C-methyl-D-erythritol; CDP-MEP, 2-phospho-4-(cytidine 5′-diphospho)-2-C-methyl-D-erythritol; cMEPP, 2-C-methyl-D-erythritol 2,4-cyclodiphosphate; HMBPP, 4-hydroxy-3-methylbut-2-enyl diphosphate; HMG-CoA, 3-Hydroxy-3-methylglutaryl-CoA; MVA, mevalonate; MVAP, mevalonate-5-phosphate; MVAPP, mevalonate-5- diphosphate; FPP, farnesyl diphosphate.

**Figure 3:**
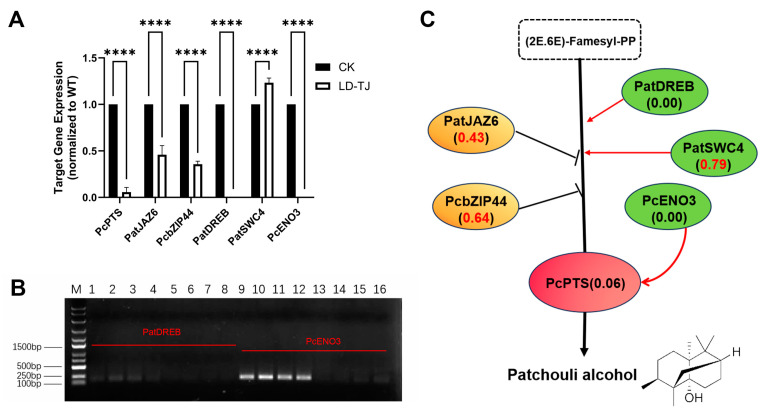
Expression profile levels of upstream regulatory genes of PTS, a key rate-limiting enzyme for PA biosynthesis. (**A**) Relative gene expression was assessed by RT-qPCR analysis. Asterisks indicate significant compared to CK controls, which were set to a value of 1. ****, *p* < 0.0001 (by two-way ANOVA with Bonferroni’s multiple-comparison test, *n* ≥ 3 independent experiments performed in triplicate). (**B**) Gene expression analysis of PatDREB and PcENO3 encoding genes by RT-PCR. M represents the DL5000 marker bands; lanes 1–4 and 9–12 represent the expression levels of the PatDREB and PcENO3 protein-encoding genes in four biological replicates of the CK group, respectively, while lanes 5–8 and 13–16 represent the expression levels of the PatDREB and PcENO3 protein-encoding genes in four biological replicates of the LD-TJ group, respectively. (**C**) Gene expression values are normalized to the control group (CK), shown as an average of three replicates. The yellow background represents negative regulators, and green represents positive regulators.

Alongside the GC-MS quantification results, we first assessed the expression levels of the *PcPTS* (*PTS*) gene, which encodes a key rate-limiting enzyme in patchouli synthesis, using real-time PCR (qPCR). The findings indicated a substantial reduction of 94% in PTS expression in LD-TJ leaves compared to CK. Additionally, the expression of the FPPS-encoding gene was markedly decreased by 79% (Figure 2A). Furthermore, we examined the expression levels of most of the key genes in the two metabolic pathways of the methylerythritol phosphate (MEP) and MVA, which are closely related to terpenoid synthesis. The results demonstrated that over 70% of gene expressions in the infected leaves exhibited a downward trend, with only four genes showing an upregulation in expression levels (Figure 2A). Notably, the expression of the MCT (2-C-methyl-D-erythritol 4-phosphate cytidylyltransferase)-encoding gene was undetectable in any of the four biological replicates of the diseased leaf samples (Figure 2B). Figure 2C quantitatively and visually represents these findings, illustrating that aside from the upregulation of four genes, the majority of gene expressions generally declined, with eight genes showing a reduction in expression by more than 50%. In addition, we investigated the expression of upstream regulators that impact the transcription of *PTS* in LD-TJ leaves compared to CK leaves. Specifically, we focused on four transcription factors: PatJAZ6, PatSWC4, PcbZIP44, and PatDREB, in addition to an interacting protein, PcENO3. In the LD-TJ group, the expression levels of patJAZ6 and pcbZIP44, which function as negative regulators of *PTS*, were reduced by over 50% compared to their expression in the CK group (Figure 3A). Among the positive regulators, the expression of patSWC4 increased by 24% (Figure 3A), while the expression of patDREB and pcENO3 were undetectable across all four biological replicates (Figure 3B). Figure 3C illustrates the specific gene expression values of these regulators relative to CK in the LD-TJ group.

### 2.3. Non-Targeted Metabolome Analysis of Volatile Compounds in LD-TJ and CK Leaves

**Figure 4:**
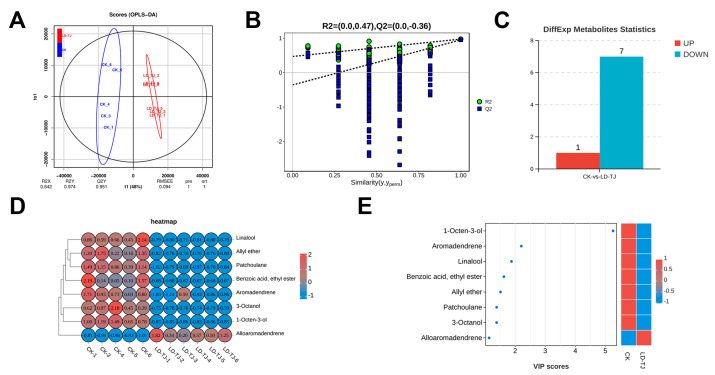
Multivariate statistical analysis and differential abundance metabolites (DAMs) in CK and LD-TJ groups were analyzed by GC-MS. (**A**) Orthogonal projection to latent structures-discriminant analysis (OPLS-DA) score plots. The black circle represents the 95% confidence interval (Hotelling’s T-squared ellipse). The parameters obtained were R^2^X = 0.642, R^2^Y = 0.974, and Q^2^Y = 0.951, indicating high reliability. R^2^X and R^2^Y denote the degree of explanation of the OPLS-DA model for the categorical variables X and Y, respectively. Q^2^Y represents the predictability of the models. RMSEE, root mean square error of estimation. (**B**) Cross-validation and permutation test of the OPLS−DA model. Green circle, R^2^; blue square, Q^2^. The y-axis represents the values of R^2^Y and Q^2^, with R^2^Y being (0.0, 0.47) and Q^2^ being (0.0, −0.36) in this result. The x-axis represents the permutation retention rate, with x = 1.0 indicating the original model’s R^2^ and Q^2^ values. Dashed lines represent the regression lines of R^2^Y and Q^2^. Q^2^ regression line intersection at y-axis ≤ 0 indicates model reliability without overfitting. (**C**) Number of DAMs in CK and LD-TJ groups. “up” and “down” indicate up- and down-regulated expressions. (**D**) Heatmap of differential metabolites. (**E**) The VIP value plots of the differential metabolites. The thresholds set were a VIP value ≥ 1 in the OPLS-DA model and a *t*-test *p*-value < 0.05.

To thoroughly examine the differences in volatile organic compound profiles between LD-TJ and CK groups, a non-targeted metabolomic analysis was conducted using headspace-solid-phase microextraction coupled with gas chromatography-mass spectrometry (HS-SPME-GC-MS). Based on all samples, electron impact (EI) ionization was used to detect 68 volatile metabolites (Table S1A). Principal component analysis (PCA) revealed that the first two principal components (PC1 and PC2) accounted for 52.9% and 13.6% of the total variance, respectively, and clearly distinguished each treatment group (Figure S1A). In contrast to PCA, OPLS-DA is a supervised method that excludes unrelated orthogonal variables, enabling the identification of more reliable metabolites. As shown in the OPLS-DA score plots, samples from different treatment groups were effectively differentiated (Figure S1B). Unlike the PCA results, the LD-TJ group exhibited tighter clustering in the OPLS-DA analysis compared to the PCA method. Additionally, the OPLS-DA results revealed that sample CK-2 in the CK group fell outside the 95% confidence interval (Hotelling’s T-squared ellipse), identifying it as an outlier (Figure S1B). A similar issue was observed in the PLS-DA model (Figure S1C). Therefore, we excluded the CK-2 sample and re-analyzed the data using the OPLS-DA model, resulting in more reliable group differentiation (Figure 4A). This OPLS-DA model exhibited R^2^X > 0.5 and both R^2^Y and Q^2^ > 0.9, indicating excellent performance and confirming metabolite responses to disease treatment. Validation using 200 randomized permutation tests revealed that all permuted Q^2^ values were lower than the original model’s Q^2^ and that the Q^2^ regression line intercept was below zero. These findings demonstrate the model’s robustness and indicate an absence of overfitting (Figure 4B). Based on the OPLS-DA results, metabolites with VIP ≥ 1 and fold changes of ≥ 2 or ≤ 0.5 were selected for differentially abundant metabolites (DAMs) analysis. This analysis identified eight significant metabolites, of which only Alloaromadendrene was significantly upregulated in the LD-TJ group (Figure 4C). The remaining seven metabolites—1-Octen-3-ol, aromadendrene, linalool, Benzoic acid ethyl ester, Allyl ether, patchoulane, and 3-Octanol—were all significantly downregulated (Figure 4D). Additionally, a VIP plot was utilized to rank the DAMs based on their contribution to distinguishing between the two groups, ordering them from highest to lowest contribution (Figure 4E).

### 2.4. Non-Targeted Metabolome Analysis of Non-Volatile Compounds in LD-TJ and CK Leaves

**Figure 5:**
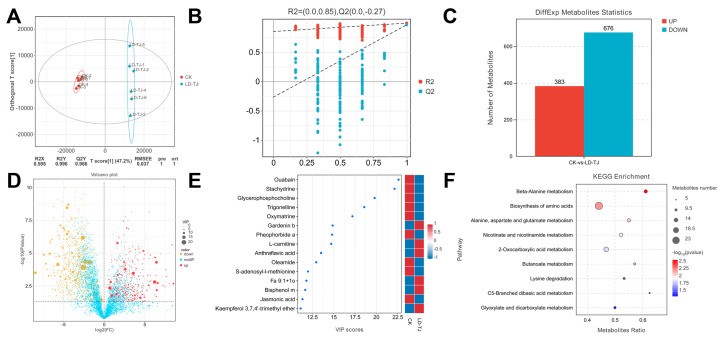
Multivariate statistical analysis and differential abundance metabolites (DAMs) in CK and LD-TJ groups were analyzed by LC-MS. (**A**) Orthogonal projection to latent structures-discriminant analysis (OPLS-DA) score plots. The parameters obtained were R^2^X = 0.595, R^2^Y = 0.996, and Q^2^Y = 0.966, indicating high reliability. R^2^X and R^2^Y denote the degree of explanation of the OPLS-DA model for the categorical variables X and Y, respectively. Q^2^Y represents the predictability of the models. RMSEE, root mean square error of estimation. (**B**) Cross-validation and permutation test of OPLS−DA model. The ordinate represents the R^2^ and Q^2^ replacement test values. Red circle, R^2^; blue circle, Q^2^. (**C**) Number of DAMs in CK and LD-TJ groups. “up” and “down” indicate up- and down-regulated expressions. (**D**) Volcano plots of metabolomic profiles. Significantly altered metabolites were selected according to VIP > 1 and *p* < 0.05 and were identified by searching the database. (**E**) The VIP value plots of the differential metabolites (the top 15 of VIP value). The thresholds set were a VIP value ≥ 1 in the OPLS-DA model and a *t*-test *p*-value < 0.05. (**F**) Significant KEGG pathways enrichment analysis bubble plot (*p*-value < 0.05). The bubble size represents the number of DAMs.

**Figure 6:**
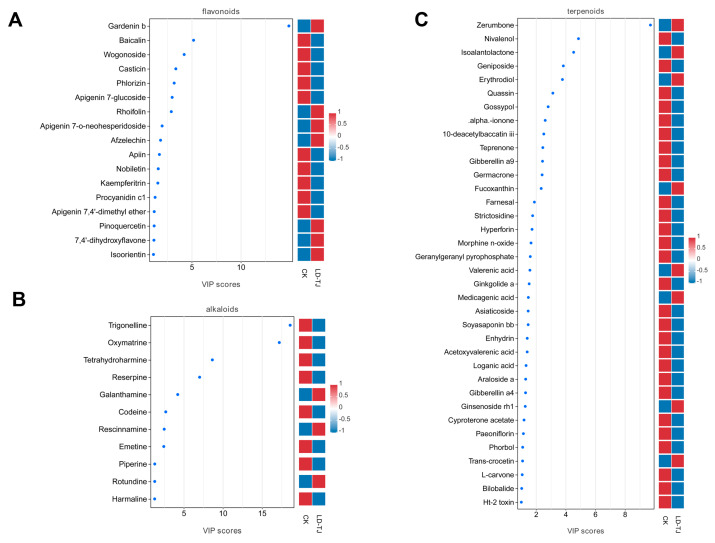
The VIP value plots of the differential metabolites of flavonoid (**A**), alkaloid (**B**), and terpenoid (**C**) components. The thresholds set were a VIP value ≥ 1 in the OPLS-DA model and a *t*-test *p*-value < 0.05.

To compare non-volatile metabolite contents in LD-TJ and CK leaves, we used LC-MS/MS untargeted metabolomics to create metabolite profiles. PCA showed that quality control (QC) samples clustered tightly in both positive (POS) and negative (NEG) ion modes, confirming data reliability (Figure S2A). The PC1 score plot distinctly separated the LD-TJ and CK groups, indicating that CLSD significantly altered the metabolite profile of patchouli leaves. In POS, PC1 and PC2 explained 35.6% and 17.8% of the variance, respectively, while in NEG, they accounted for 36.5% and 17.7%, respectively (Figure S2A). Utilizing databases such as MassBank and METLIN, we identified 2379 metabolites in the POS and 3414 metabolites in the NEG (Tables S2 and S3). Hierarchical clustering analyses showed distinguishable metabolite expression patterns between the CK and LD-TJ groups in both POS and NEG modes (Figure S2B). According to the unsupervised nature of the PCA model, OPLS-DA was used. OPLS-DA and PCA were satisfactory in both LD-TJ vs. CK group separation, where the CK group showed less within-group variance as compared to LD-TJ. The OPLS-DA model parameters revealed that R^2^X values exceeded 0.5, while both R^2^Y and Q^2^Y values were greater than 0.9, indicating excellent predictive ability (Figure 5A). Additionally, permutation tests demonstrated that the OPLS-DA model achieved R^2^ and Q^2^ values of 0.85 and −0.27, respectively, thereby confirming the model’s robustness and absence of overfitting (Figure 5B). Then we combined the VIP values derived from the OPLS-DA with the *p*-values obtained from univariate *t*-tests to identify DAMs between the CK and LD-TJ groups. Our analysis revealed that, in comparison to the LD-TJ group, the CK group exhibited 383 upregulated and 676 downregulated metabolites (Figure 5C and Table S4). This differential expression was illustrated in the volcano plot (Figure 5D), where red dots denoted upregulated metabolites and yellow dots denoted downregulated metabolites in the LD-TJ group relative to the CK group. The size of the dots correlated with the VIP values from the OPLS-DA model, with larger dots indicating higher VIP values. Considering that the higher the VIP value, the greater the contribution of the metabolite to the differentiation of the sample, we selected the top 15 differential metabolites with VIP values in each of the two modes according to the VIP value from large to small (Figure 5E). As illustrated in Figure 5E, nine metabolites exhibited significant up-regulation in the CK group relative to the LD-TJ group, whereas six metabolites demonstrated a marked increase in abundance within the LD-TJ group. To elucidate the biological processes influenced by these DAMs, we performed a KEGG pathway analysis. In the KEGG enrichment assay, nine pathways showed significant differences between CK and LD-TJ groups, namely, “beta-Alanine metabolism”, “Biosynthesis of amino acids”, “Alanine, aspartate and glutamate metabolism”, “Nicotinate and nicotinamide metabolism”, “2-Oxocarboxylic acid metabolism”, “Butanoate metabolism”, “Lysine degradation”, “C5-Branched dibasic acid metabolism”, “Glyoxylate and dicarboxylate metabolism” (Figure 5F). For certain secondary metabolites that exhibited significant pharmacological properties, including flavonoids, alkaloids, and terpenoids, VIP plots were employed to illustrate metabolites that demonstrated substantial differences across various class groups (Figure 6).

## 3. Discussion

Currently, the impact of diseases on the quality and metabolic products of herbal medicines has not been adequately studied [35,36]. Patchouli alcohol, a principal component of essential oil and a critical quality indicator in the Chinese Pharmacopoeia, is essential for the quality of Pogostemonis Herba [26]. In light of this, the present study first investigated the effect of *C. cassiicola* on the patchouli alcohol content in *P. cablin* leaves using GC-MS. The 51% reduction in patchouli alcohol content in CLSD-infected leaves demonstrated a significant disruption of terpenoid biosynthesis, which is critical for the medicinal quality of Pogostemonis Herba. This decline correlates with the suppression of key genes in the MEP and MVA pathways, such as PTS (94% reduction) and FPPS (79% reduction), and over 70% of the MEP and MVA pathways-associated genes exhibited diminished expression in CLS-infected leaves, with the MCT gene being completely undetectable. Similar disruptions have been observed in *Santalum album*, where biotic stresses such as *Fusarium oxysporum* infection caused changes in the expression of terpene synthase genes, greatly impacting sesquiterpenoid production [37]. Furthermore, miRNA-mediated regulation of terpenoid biosynthetic pathways has been implicated in pathogen-induced metabolic reprogramming, as seen in *Persicaria minor* under *F. oxysporum* stress [38]. These findings suggest that *C. cassiicola* may employ similar strategies to suppress terpenoid biosynthesis through transcriptional and post-transcriptional mechanisms. Such disruptions not only compromise the plant’s defense mechanisms but also reduce the therapeutic value of terpenoid-rich essential oils [39].

The intricate alterations in transcriptional regulatory networks underscore the complexity of host-pathogen interactions and their profound influence on plant defense mechanisms. In patchouli, the unexpected downregulation of negative regulators such as PatJAZ6 and PcbZIP44, alongside the modest upregulation of PatSWC4, indicates an attempted but ultimately ineffective compensatory response aimed at sustaining patchouli alcohol biosynthesis. Furthermore, the complete suppression of positive regulators such as PatDREB and PcENO3 likely represented a critical vulnerability in the plant’s defense system, as their absence compromised the ability to mount effective immune responses. Similar disruptions in transcriptional networks have been reported in rice during *Magnaporthe oryzae* infection, where key defense-related genes are silenced [40]. Moreover, *Pseudomonas syringae* has been shown to increase plant susceptibility to infection by disrupting auxin synthesis or transport mediated by PIN1 in *Arabidopsis thaliana* [41]. Such pathogen-induced reprogramming often involves the hijacking of transcriptional hubs, targeting essential transcription factors such as WRKYs and NACs that play central roles in immune signaling [42]. This interference leads to extensive transcriptional silencing, suppression of terpenoid biosynthesis, and a reallocation of metabolic resources, ultimately weakening plant health and reducing the production of secondary metabolites critical for medicinal applications. In summary, this study demonstrated that the disease significantly disrupted both the biosynthesis of patchouli alcohol and its associated metabolic network, ultimately affecting the quality of the herbal medicine. These findings highlight the necessity for further research into pathogen effectors and their specific roles in manipulating host metabolic pathways.

It is well established that the evaluation of Chinese herbs for quality extends beyond a single indicator, necessitating the assessment of the main active ingredients. Non-targeted metabolome analysis stands out as a crucial method in medicinal plant research due to its ability to provide a comprehensive overview of metabolites, enhance understanding of complex biochemical interactions, ensure product quality, and adapt across various species and conditions [10,43,44]. In this study, HS-SPME-GC-MS metabolomic analysis revealed that CLSD induced widespread alterations in volatile organic compounds (VOCs), with a clear trend toward suppression rather than enhancement. The predominant downregulation of VOCs, with 7 out of 8 DAMs being reduced, included three terpenoids—aromadendrene, linalool, and patchoulane—which were directly linked to the terpenoid biosynthesis pathway. The significant reduction in their levels was closely associated with the downregulation of 70% of genes involved in the MEP and MVA pathways, as shown in Figure 2. Notably, the synthesis of both patchoulane and patchouli alcohol requires the catalytic activity of the PcPTS enzyme [45], whose significantly reduced expression directly contributes to the sharp decline in their accumulation. In contrast, the unique upregulation of Alloaromadendrene warrants further investigation, as it may represent either a specific plant defense response or a pathogen-induced manipulation of plant metabolism. Although current research on the precise biological function of Alloaromadendrene in plants remains limited, existing studies have demonstrated its antibacterial properties [46], suggesting a potential role in plant defense mechanisms. This finding lays the groundwork for future exploration of the biological functions of Alloaromadendrene.

Considering that patchouli is known to contain a diverse range of non-volatile compounds, including flavonoids, terpenoids, and alkaloids, which possess antioxidant, anti-inflammatory, and anticancer properties [23], this study employed LC-MS/MS-based untargeted metabolomics to investigate the metabolic changes induced by CLSD. The analysis revealed extensive metabolic reprogramming in non-volatile metabolites, with a marked predominance of downregulated compounds (676 downregulated versus 383 upregulated), indicating substantial disruption of both primary and secondary metabolic pathways. Notably, the enrichment of amino acid metabolism and carboxylic acid metabolism pathways suggested that CLSD not only impacted specialized metabolism but also perturbed fundamental cellular processes. These observations align with findings in other plant-pathogen systems, where pathogen infection triggers widespread metabolic shifts [47]. Further, the significant enrichment of beta-alanine metabolism and nicotinate/nicotinamide metabolism pathways pointed to potential disruptions in cellular energy homeostasis and stress response mechanisms. This is consistent with previous reports in medicinal plants under pathogen stress, where alterations in primary metabolic pathways often precede changes in specialized metabolite production [48]. Among the top 15 differential metabolites identified based on VIP scores, several key metabolites involved in phospholipid remodeling, chlorophyll degradation, phosphate starvation responses, and cellular energy metabolism were significantly downregulated in the CLS-infected group (LD-TJ). These included glycerophosphocholine, pheophorbide a, and S-adenosyl-l-methionine [49,50]. Conversely, metabolites associated with plant defense and antimicrobial activity, such as L-carnitine and polygodial, exhibited significant upregulation following pathogen infection [51,52]. Of particular interest, jasmonic acid (JA) levels were significantly reduced in the LD-TJ group, consistent with our previous findings that JA regulates PTS expression through interactions between PatSWC4 and PatJAZ4 [29]. This reduction in JA content may play a critical role in the downregulation of terpenoid biosynthesis, further contributing to the decline in patchouli alcohol production. In summary, the comprehensive metabolomic analysis revealed profound alterations in metabolite profiles between healthy and diseased leaves, highlighting the detrimental impact of CLSD on the metabolic network and overall quality of patchouli. These findings provide valuable insights into the metabolic mechanisms underlying disease-induced quality deterioration and lay the foundation for future research into targeted interventions.

In summary, the comprehensive metabolomic analysis revealed significant alterations in metabolite profiles between healthy and diseased leaves, highlighting the negative impact of leaf spot disease on the quality of *P. cablin*. Regarding the significant reduction of patchouli alcohol, which is the key quality indicator of Pogostemonis Herba according to Chinese Pharmacopoeia, our integrated analysis reveals several contributing factors: the severe suppression of the terminal enzyme PTS, disruption of upstream precursor supply through downregulation of FPPS and most MEP/MVA pathway genes, silencing of positive transcriptional regulators (PatDREB and PcENO3), and reduction in jasmonic acid levels that normally regulate terpenoid biosynthesis. These findings underscore how pathogen infection compromises not only plant health but also the medicinal value of herbs through specific disruption of key biosynthetic pathways.

## 4. Materials and Methods

### 4.1. Fungal Infection Assay

The *C. cassiicola* LD-TJ strain used in this experiment was acquired from our laboratory and stored at the Research Center of Chinese Herbal Resource Science and Engineering at Guangzhou University of Chinese Medicine [34]. The LD-TJ strain was cultured on potato dextrose agar medium (composition: 200 g potato infusion, 20 g dextrose, 15 g agar per liter) at 28 °C for 5 to 7 days. Sporulation was induced by exposing the cultures to near-UV light for 80 min, followed by two days of incubation at 28 °C with >90% RH humidity. Subsequently, conidial suspensions were prepared and adjusted to a concentration of 1 × 10^6^ CFU/mL using sterile distilled water. Three-month-old *P. cablin* seedlings were inoculated by spraying 30 mL of LD-TJ spore suspension onto their leaves and subsequently incubated at 28 °C with artificial lighting provided for a 16-h light cycle and 80% relative humidity, while potted plants treated solely with sterile water served as controls. Seven days post-inoculation, the leaves were harvested, and all samples were immediately stored at −80 °C until further use.

### 4.2. Quantitative Analysis of Patchouli Alcohol by GC-MS

Quantification of patchouli alcohol levels was performed based on the protocol described by Wang et al. with modifications [23]. Leaf tissues were ground with a pestle to a fine powder. A 200 mg quantity of each sample powder was weighed, and 1.5 mL of hexane was added into a centrifuge tube. The mixture underwent sonication for a duration of 30 min at a frequency of 60 Hz, followed by heating in a water bath at 56 °C for a period of 1 h. Subsequent to centrifugation at 10,000 rpm, the resulting supernatant was extracted and filtered through a 0.22-µm organic membrane to create the test solution for gas chromatography-mass spectrometry (GC-MS) analysis utilizing an Agilent 7890B gas chromatograph equipped with a 5977A inert Mass Selective Detector (Agilent, Santa Clara, CA, USA). In the gas chromatograph, a capillary column with a diameter of 250 mm and a thickness of 0.25 mm was used. The instrument was programmed to initiate at a temperature of 50 °C and remain constant for a duration of 2 min. Subsequently, the oven temperature was ramped up to 130 °C at a rate of 20 °C/min, followed by an increase to 150 °C at a rate of 2 °C/min. Following a constant temperature of 150 °C for 5 min, the temperature was raised by 20 °C per minute to 230 °C. The injection volume was set at 1 µL, with the injection port temperature maintained at 230 °C. The carrier gas, helium, flowed at a rate of 1 mL/min. Split injection mode was utilized with a split ratio of 1:10. Statistical analyses were performed using Prism software version 10.0.1 from GraphPad. Comparisons between means were performed with Student’s *t*-test. Each experiment was biologically repeated three times.

For a standard curve, a total of 10 mg of patchouli alcohol standard (GC ≥ 98%, NanTong FeiYu, Nantong, China, IP0680) was placed in a volumetric flask, and the solution volume was made up to 10 mL with hexane. The mother liquor was diluted, respectively, to 10, 20, 40, 80, 100, and 200 times with hexane and then analyzed by the method described above.

### 4.3. RNA Extraction and Quantitative Real-Time PCR (qPCR) Assays

RNA extraction was conducted using the HiPure Plant RNA Mini Kit C (Magen, Guangzhou, China), as previously described [32]. Thereafter, the HiScript^®^ III RT SuperMix for qPCR (+gDNA wiper) (Vazyme, Nanjing, China) was used to synthesize the cDNA for each RNA sample. In addition, the qPCR assay was performed using the ChamQ Universal SYBR qPCR Master Mix (Vazyme, China) on a CFX96 Real-Time PCR Detection System (Bio-Rad, Hercules, CA, USA), according to the manufacturer’s protocol. The internal reference gene utilized was Pc18S, and the relative gene expression levels were determined through the ΔΔC_T_ method in accordance with the Minimum Information for Publication of Quantitative Real-Time PCR Experiments (MIQE) guidelines. The primers for the SYBR green quantitative polymerase chain reaction (qPCR) were designed using Beacon designer software version 8.0 (Premier Biosoft International, San Francisco, CA, USA) and are detailed in Table S5. Statistical analyses were performed using Prism software (GraphPad Prism version 10.0.1), with statistical significance assessed via two-way analysis of variance (ANOVA) and Bonferroni’s multiple comparisons test to compare each cell mean with the control cell mean within the respective row. Each experiment was biologically repeated three times.

### 4.4. Non-Targeted Metabolome Analysis of Volatile Compounds by HS-SPME-GC-MS

Gas chromatography–mass spectrometry (GC–MS) analyses were performed using a Thermo Trace 1300 gas chromatograph coupled with an ISQ7000 mass spectrometer (Thermo Fisher Scientific Inc., Waltham, MA, USA). A DB-WAX column (30 m × 0.25 mm × 0.25 µm) was utilized for chromatographic separation. For sample preparation, 0.2 g of each sample was placed into a headspace vial, and 500 ng of 2-methyl-3-heptanone was added as an internal standard. The chromatographic conditions were set as follows: injection temperature of 230 °C, split ratio of 10:1, and helium (99.999%) as the carrier gas at a flow rate of 1.0 mL/min. The column temperature was initially maintained at 50 °C for 4 min, then ramped at a rate of 5 °C/min to 230 °C and held for an additional 5 min. Both the interface and ion source temperatures were maintained at 230 °C. Electron ionization (EI) was conducted at 70 eV, and mass spectra were acquired in full scan mode over a mass range of 33–550 *m*/*z*. For solid-phase microextraction (SPME), a CTC triple autosampler was employed with a 65 μm PDMS/DVB (1 cm) extraction fiber. The extraction conditions were as follows: samples were equilibrated at 50 °C with shaking at 250 rpm for 15 min, followed by SPME extraction for 30 min. Desorption was carried out for 5 min, and the GC run time was set to 50 min. Data processing was performed using Chromeleon software (version 7.0), utilizing the NIST 2017 library for feature peak extraction. Background values from relatively stable baselines were subtracted, and a peak width of 0.1 was set. Peak integration was carried out by selecting the compound with the highest similarity score. Qualitative ion calibration was applied, and unidentified peaks were removed during data organization. Metabolite identification was performed using the Kovats index of each of the detected peaks, calculated from the retention times of a mixture of C-7 to C-40 alkanes injected at 100 µg/mL under the same conditions as the patchouli samples (Table S1B). The *retention index* (*RI*) was determined by calibration with standard alkanes. The RI calculation is performed using the following formula:

$$RI=100\times n+\frac{100\times (t_{a}-t_{n})}{t_{n+1}-t_{n}}$$

The final results were organized into a two-dimensional data matrix, encompassing retention time, *RI* values, CAS numbers, classification information, and other details.

### 4.5. Non-Targeted Metabolome Analysis of Non-Volatile Compounds by LC-MS/MS

The LC-MS/MS methodology was adapted from our previous report with modifications [23]. Samples were frozen in liquid nitrogen and ground with a pestle and mortar. For extraction, 100 mg of each sample was mixed with 1 mL of cold methanol/acetonitrile/water (2:2:1, *v*/*v*/*v*), sonicated at low temperature for two 30-min intervals, and centrifuged at 14,000× *g* for 20 min at 4 °C. The supernatant was dried in a vacuum centrifuge and re-dissolved in 100 µL of acetonitrile/water (1:1, *v*/*v*) before LC-MS/MS analysis. Quality control (QC) samples were included to ensure data reliability. LC-MS/MS was performed using an Agilent 1290 Infinity UHPLC system coupled to an AB Sciex TripleTOF 6600 mass spectrometer (Agilent, Santa Clara, CA, USA). Separation was achieved on a Waters ACQUITY UPLC BEH HILIC (Waters, Milford, MA, USA) column (2.1 mm × 100 mm, 1.7 µm) with a mobile phase of 25 mM ammonium acetate and 25 mM ammonium hydroxide in water (A) and acetonitrile (B). The gradient started at 85% B for 1 min, decreased to 65% B over 11 min, then to 40% B in 0.1 min, held for 4 min, and returned to 85% B within 0.1 min, including a 5-min re-equilibration. ESI source settings were Gas1 and Gas2 at 60, CUR at 30, source temperature at 600 °C, and ISVF at ±5500 V. MS acquisition scanned *m*/*z* 60–1000 Da (MS) and 25–1000 Da (MS/MS) with IDA in high sensitivity mode. Data were processed with ProteoWizard MSConvert (version 3.0.9974) and XCMS platform, followed by CAMERA in R (version 3.0.2) for isotope and adduct annotation. Metabolites were identified by accurate *m*/*z* (<10 ppm) and MS/MS comparison to an in-house database. Missing values were imputed using K-Nearest Neighbors, outliers removed, and peak areas normalized for uniformity.

### 4.6. Statistical Treatment of Data

The metabolomic data of volatile and non-volatile compounds was conducted in the same manner as follows. Principal component analysis (PCA) was conducted using the R language gmodels (version 2.18.1). The reliability of the results is directly proportional to the density of the QC sample distribution. Multivariate statistical analysis was performed by Orthogonal Partial Least Squares (OPLS), which was applied in comparison groups using R package models (http://www.r-project.org/, accessed on 26 June 2024). The OPLS-DA model was further validated by cross-validation and permutation test [53]. In the analysis of differential metabolites, a variable importance in projection (VIP) score from an OPLS-DA model was utilized to prioritize metabolites that exhibited significant distinctions between two groups. A VIP threshold of 1 was established for this purpose. Additionally, a *t*-test was employed as a univariate analysis to identify differential metabolites, with those exhibiting a *t*-test *p*-value < 0.05 and a VIP score ≥ 1 being deemed as such. The abundance of these differential metabolites within the same group was standardized using z-scores. Subsequently, the VIP scores derived from the OPLS-DA model were utilized to generate graphical representations. The top 15 metabolites are shown in the variable importance in projection (VIP) score plot in descending order. Metabolites were mapped to KEGG metabolic pathways for annotation and enrichment analysis.

## 5. Conclusions

**Figure 7:**
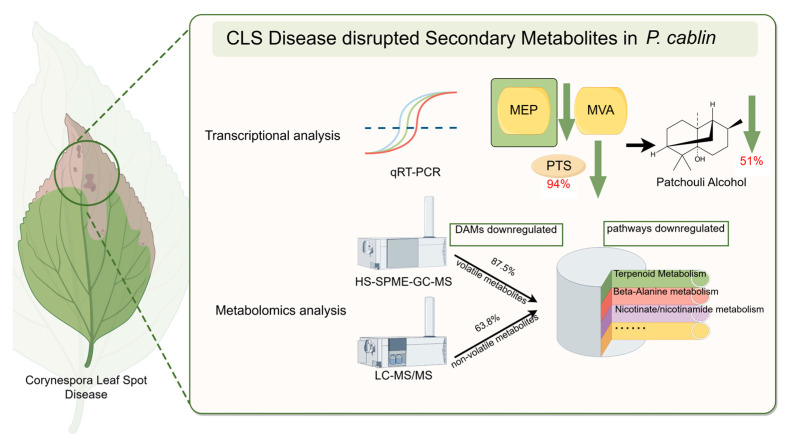
Transcriptional and metabolic alterations in *P. cablin* leaves infected with Corynespora leaf spot disease (CLSD). Integrated transcriptional and untargeted metabolomic analyses revealed that CLSD significantly reduced patchouli alcohol content by 51% in *P. cablin* leaves, strongly associated with a 94% downregulation of the essential PcPTS biosynthetic gene. Comprehensive metabolomic profiling demonstrated extensive disruptions across both volatile and non-volatile metabolites, with 87.5% of differentially abundant metabolites (DAMs) among volatiles and 63.8% among non-volatiles showing decreased concentrations. Pathway analysis further identified predominant downregulation of key metabolic processes, particularly terpenoid metabolism, beta-alanine metabolism, and nicotinate/nicotinamide metabolism.

This study highlights the significant adverse effects of *C. cassiicola*-induced CLSD on *P. cablin*, a commercially important industrial aromatic crop. Our findings reveal a 51% reduction in patchouli alcohol content, the primary industrial compound, in diseased leaves compared to healthy controls. This reduction is strongly associated with the significant downregulation of essential biosynthetic genes, including a 94% drop in the PcPTS gene and a 79% decrease in the FPPS gene expression, both vital for terpenoid production. Comprehensive metabolomic analyses uncovered extensive disruptions in both volatile and non-volatile metabolites, with a predominant downregulation of essential compounds and key metabolic pathways such as terpenoid metabolism, beta-alanine metabolism, and nicotinate/nicotinamide metabolism (Figure 7). These metabolic changes not only impair the plant’s ability to produce valuable industrial compounds but also indicate broader metabolic instability caused by CLSD. Additionally, the downregulation of crucial transcription factors and interacting proteins suggests a complex mechanism by which *C. cassiicola* manipulates host metabolic processes to its advantage. These findings emphasize the urgent need to develop disease-resistant *P. cablin* cultivars through genetic and metabolic engineering strategies to ensure the sustainability and productivity of this economically important crop. Furthermore, understanding the molecular basis of disease resistance can guide integrated crop management practices to mitigate the impact of biotic stresses on industrial crop quality and yield.

## Data Availability

The original contributions presented in this study are included in the article/Supplementary Materials. Further inquiries can be directed to the corresponding author(s).

## Supplementary Materials

The supporting information can be downloaded at https://www.mdpi.com/article/10.3390/ijms26083680/s1.
