# Supplementary material for: Metabolomic Profiling of Pogostemon cablin Reveals Disruption of Secondary Metabolite Biosynthesis Induced by Corynespora cassiicola Infection
Source: Int J Mol Sci. 2025 Apr 13;26(8):3680. doi: 10.3390/ijms26083680 (PMC12027274; doi:10.3390/ijms26083680)
Supplement: Supplementary file 1 [file ijms-26-03680-s001.zip › supplementary figures.pdf]

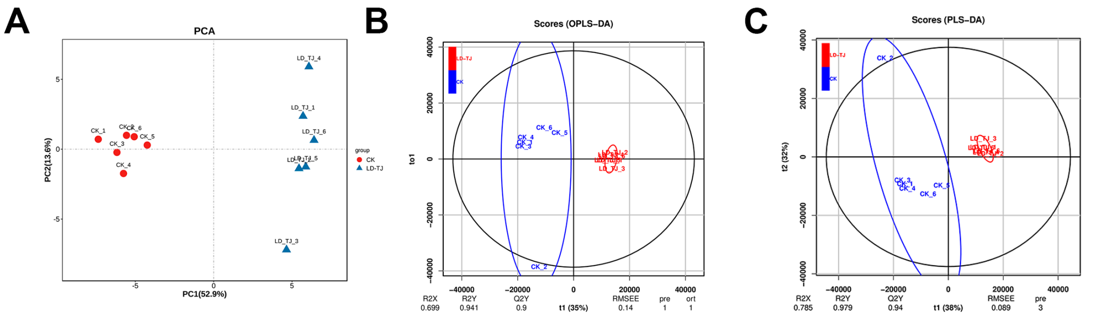

Figure S1. Multivariate statistical analysis of volatiles metabolite profiles between CK and LD-TJ groups. (A) PCA plot exhibiting the result of quality control of metabolome data. (B) Orthogonal projection to latent structures-discriminant analysis (OPLS-DA) score plots. The black circle represents the 95% confidence interval (Hotelling's T-squared ellipse). The parameters obtained were $R^2X = 0.699$, $R^2Y = 0.941$, and $Q^2Y = 0.9$. R^2X and R^2Y denote the degree of explanation of the OPLS-DA model for the categorical variables X and Y, respectively. Q^2Y represents the predictability of the models. RMSEE, root mean square error of estimation. (C) Orthogonal PLS-DA Analysis (OPLS-DA) score plots. The black circle represents the 95% confidence interval (Hotelling's T-squared ellipse). The parameters obtained were $R^2X = 0.785$, $R^2Y = 0.979$, and $Q^2Y = 0.94$. R^2X and R^2Y denote the degree of explanation of the PLS-DA model for the categorical variables X and Y, respectively. Q^2Y represents the predictability of the models. RMSEE, root mean square error of estimation.

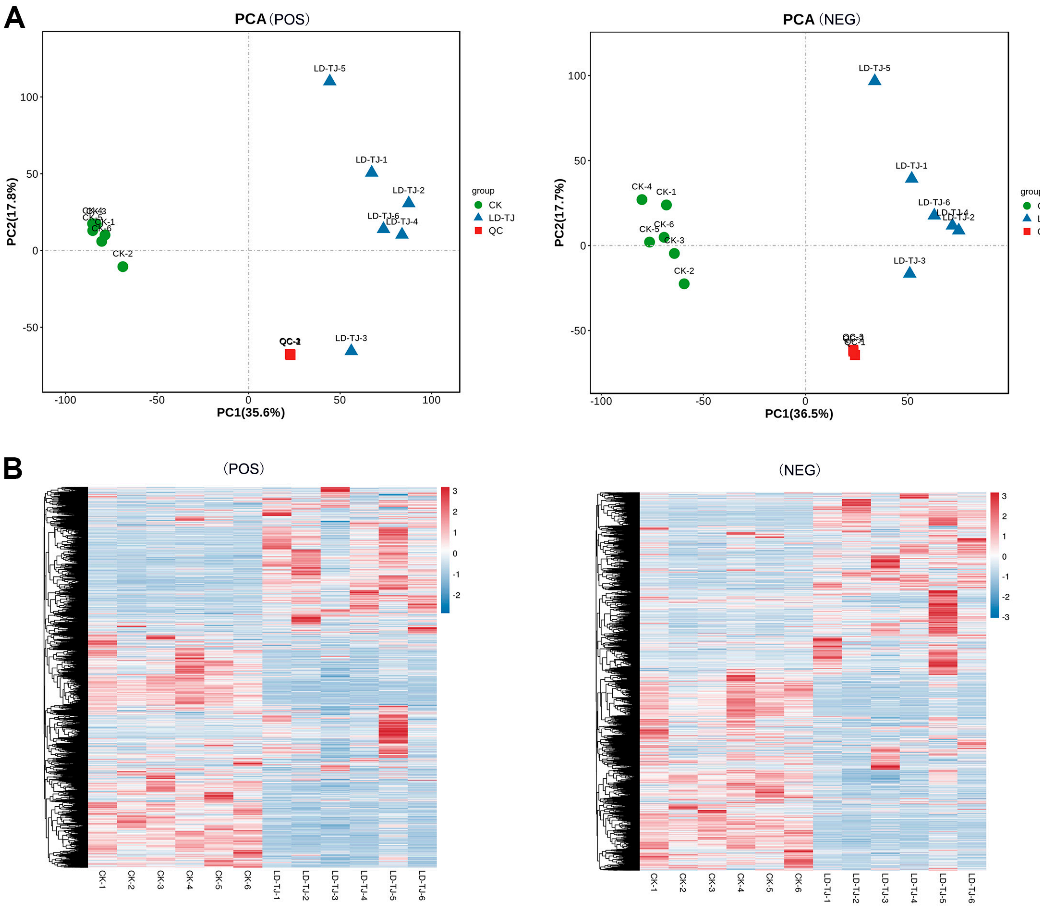

Figure S2. Quality control and heat map of untargeted metabolomics features of non-volatiles

metabolites. (A) PCA plot exhibiting the result of quality control of metabolome data. The densely distributed of QC samples indicates the high reliability of the metabolome data. (B) The correlation matrix of gene expression among 6 independent biological experiments of different groups.
